# Shepherd's Purse Polyphenols Exert Its Anti-Inflammatory and Antioxidative Effects Associated with Suppressing MAPK and NF-*κ*B Pathways and Heme Oxygenase-1 Activation

**DOI:** 10.1155/2019/7202695

**Published:** 2019-01-13

**Authors:** Jinming Peng, Tianyong Hu, Jin Li, Jing Du, Kerui Zhu, Baohui Cheng, Kaikai Li

**Affiliations:** ^1^College of Food Science and Technology, Huazhong Agricultural University, Wuhan 430070, China; ^2^Shenzhen Key Laboratory of ENT, Longgang ENT Hospital & Institute of ENT, Shenzhen 518172, China; ^3^Key Laboratory of Environment Correlative Food Science (Huazhong Agricultural University), Ministry of Education, Wuhan 430070, China

## Abstract

Shepherd's purse (*Capsella bursa-pastoris* (L.) Medik.), a wild herb as a traditional herbal medicine, has been proved with multiple healthy benefits. In this study, the chemical constituents of shepherd's purse were identified by UPLC-QTOF-MS/MS. The antioxidative and anti-inflammatory potential of shepherd's purse extract (SPE) were also investigated applying lipopolysaccharide- (LPS-) induced inflammation in RAW 264.7 macrophages and a carrageenan-induced mice paw edema model. Twenty-four chemical compounds were identified mainly including phenolic acids and flavonoids. The data also indicated SPE inhibited the productions of NO, PGE_2_, TNF-*α*, and IL-6 stimulated with LPS. In addition, SPE inhibited the increase of reactive oxygen species (ROS) and upregulated the expression of heme oxygenase-1 (HO-1). We further found that SPE inhibited the phosphorylation of P38 MAPK and activation of NF-*κ*B. *In vivo* mice model also indicated that SPE showed strong antioxidative and anti-inflammatory activity.

## 1. Introduction

Inflammation which existed in obesity, elder bodies, is accompany with many diverse chronic diseases, such as insulin resistance, type 2 diabetes, vascular disease, chronic renal failure, several cancers, endocrine [1–3]. To counteract this chronic inflammatory status, nonsteroidal anti-inflammatory drugs (NSAIDs) were usually proposed as a treatment strategy [4]. However, the side effects associated with long-term use of NSAIDs and steroids stimulate the development of novel anti-inflammatory therapies [5, 6]. Thus, the functional foods, for instance, some edible wild herbs, which with special health benefits, unique flavor, and also with high nutritional values, may be a good choice for improvement of the chronic low-grade inflammation and its related diseases. More importantly, these functional foods also showed higher biosafety and also can be easily and well accepted.

**Figure 1:**
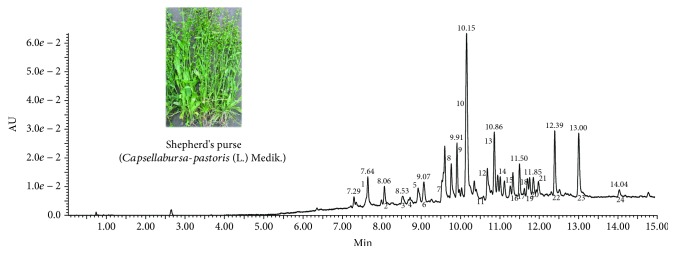
The picture of shepherd's purse and DAD chromatogram at 320 nm of shepherd's purse extracts.

Shepherd's purse (*Capsella bursa-pastoris* (L.) Medik.), a wild herb (Figure 1) with high nutritional value and has been eaten raw or cooked as a vegetable for thousands of years in many countries, is getting more and more attention. Shepherd's purse has been used as traditional herbal medicine for a long history which has been recorded in TCM ancient books “Ben Cao Gang Mu,” “Ming Yi Bie Lu,” and so on. Previous studies found that shepherd's purse contained a wide range of chemicals including flavonoids, alkaloids, polypeptides, choline, acetylcholine, histamine, tyramine, fatty acids, sterols, organic acids, amino acids, sulforaphane, many trace elements, vitamins, and many other compounds [7–11]. Furthermore, pharmacological studies also proved that shepherd's purse with various bioactivities, including anti-inflammatory, antioxidative, antiallergic, AChE inhibitory activity, and anticancer effects in previous studies [12–16]. Choi et al. prepared a sulforaphane-containing solution component from shepherd's purse and found it had significant anti-inflammatory activity [13]. Lan et al. found that the EtOAc extract of *Capsella bursa-pastoris* which with apigenin-7-O-*β*-D-glucopyranoside, luteolin-7-O-*β*-D glucopyranoside, *α*-adenosine, and uridine showed stronger anti-inflammatory activities in carrageenan-induced paw edema experiment and egg-albumin-induced inflammation experiment [17]. Even though little studies found shepherd's purse with anti-inflammatory activity, the chemical composition, antioxidative and anti-inflammatory activities of the extract of shepherd's purse, and its underlying mechanisms have not been systematically studied. Therefore, the aim of the present study was to systematically investigate the chemical composition, anti-oxidative and anti-inflammatory activities of shepherd's purse extract, and their underlying mechanisms using LPS-induced RAW 264.7 cells and an *in vivo* carrageenan-induced mouse paw edema model.

## 2. Material and Methods

### 2.1. Plant Materials and Preparation of Shepherd's Purse Extracts (SPE)

Fresh shepherd's purse was collected from Xiaogan, Hubei province of China, in March 2017. The specimen of the whole plant was deposited in College of Food Science and Technology in Huazhong Agricultural University (the voucher specimen number: 2016-02). The raw materials were dry in the shade and then were pulverized with a grinder. For extraction, 100 g raw materials were soaked with 2000 mL 95% ethanol at 100°C for 1 h for twice. The extract solution was combined and concentrated under reduced pressure and then freeze-dried using a vacuum freeze drying. The yield of extract was about 12.8% (*w*/*w*). The extracts were stored at −20°C for further use.

### 2.2. UPLC-QTOF-MS/MS System and Conditions

Chemical analysis of the SPE was performed by UPLC-QTOF-MS/MS analysis that was equipped with Waters Acquity UPLC system and MS system (Waters Corp., MA, USA). The UPLC analysis was performed with an Acquity UPLC BEH C18 column (2.1 × 100 mm, 1.7 *μ*m). The mobile phases composed of water with 0.01% formic acid (A) and methanol (B); the elution was performed with a gradient procedure according to the following conditions: 0-0.5 min, 1% B; 0.5-30 min, 1% B -99% B, with a flow rate of 0.4 mL/min. 1 mg/mL of SPE in ethanol was prepared and filtered through 0.22 *μ*m nylon micropore membranes prior to use. The injection volume was 1 *μ*L. Parameters for ESI MS are as follows: negative mode; source temperature 120°C; desolvation gas flow 800 L/h; desolvation temperature 450°C; cone gas flow 50 L/h; sampling cone and capillary voltages were 30 and 2500 V, respectively. A scan ranges from *m/z* 100 to 1500 were applied.

### 2.3. Antioxidant Activity of SPE

The radical scavenging ability of SPE was evaluated using ABTS assay. The stock solution of ABTS^+^ was prepared by admixing ABTS (7 mM) with K_2_S_2_O_8_ solution. To obtain the ABTS^+^ working solution, the above stock solution was further diluted with water until the acceptable absorbance (0.7 ± 0.02) achieved at 734 nm. Ascorbic acid (Vc) was selected as a positive control and Vc equivalent antioxidant capacity was calculated. 10 *μ*L sample with different concentration and 200 *μ*L of the working solution were mixed thoroughly, incubated for about 10 min, and the absorbances were determined at 734 nm using a microplate reader.

### 2.4. Cell Culture

The mouse macrophage cell line RAW 264.7 (ATCC, USA) was grown in DMEM culture medium (ATCC, USA) supplemented with 10% fetal bovine serum (FBS) (Gibco, USA) in a 5% CO_2_ humidified incubator at 37°C.

### 2.5. Cell Viability Assay

RAW 264.7 macrophages were seeded with a density of 4 × 10^3^ cells/well into a 96-well plate. After incubation overnight, the cells were treated with SPE (0-320 *μ*g/mL) and LPS for 20 h. Then, 20 *μ*L of 5 mg/mL of methylthiazole tetrazolium (MTT) was added into each well and then incubated for another 4 h. After that, the supernatant was discarded and 100 *μ*L DMSO was added. Plates were shaken for 1 min and the absorbance was measured at 570 nm using a microplate reader (Thermo Fisher, USA).

### 2.6. Determination of NO and Proinflammatory Cytokines

RAW 264.7 macrophages were treated as previously described [18]. Briefly, the cells were stimulated with 1 *μ*g/mL of LPS with or without SPE for 16 h. The cell-free supernatant was collected with different treatment times (1, 2, 4, 8, and 16 h). NO concentration was measured using Griess reagent (Sigma, USA) and NaNO_2_ were applied as standard. The contents of PGE_2_, TNF-*α*, and IL-6 were measured using specific ELISA kit (Cayman Chemical, Ann Arbor, Michigan, USA; R&D, Minneapolis, MN, USA) according to the manufacturer's guidelines.

### 2.7. Measurements of ROS Production

The intracellular generation of ROS was determined using a 2′, 7′-dichlorofluorescein diacetate (DCFH-DA) as previously described [18]. RAW 264.7 macrophage cells were first incubated with LPS and SPE for 16 h; after that, cells were treated with 20 *μ*M DCFH-DA at 37°C for 30 min in the dark. After that, DCF fluorescence intensity was measured by microplate fluorometer at wavelengths of 488 nm (excitation) and 535 nm (emission).

### 2.8. Western Blot Analysis

The treatment method was the same as described above. For iNOS, COX 2, and HO-1 determination, macrophages were stimulated with 1 *μ*g/mL LPS and SPE for 16 h. For signaling molecule analysis (NF-*κ*B and MAP kinase signaling), cells were treated with 1 *μ*g/mL LPS and SPE for just one hour. Protein samples with or without MG132, a proteasome inhibitor, were also collected for 0.25, 0.5, and 1 h. After that, the cells were harvested and protein was collected. The detailed information of the western blot method was the same as previous reports [19]. The blots were detected using enhanced chemiluminescence assay kit (GE Healthcare, UK) and visualized by the chemiluminescent method (BioRad Laboratories, Hercules, CA, USA). *β*-Actin was used as a control.

### 2.9. Carrageenan-Induced Mouse Hind Paw Edema

C57BL/6 mice (20-22 g) were obtained from Laboratory Animal Center of Huazhong Agricultural University (Wuhan, China). All the procedures were approved by the Experimental Animal Review Committee of Huazhong Agricultural University of China. First, 100-400 mg/kg of SPE were administered orally; after 1 h, 30 *μ*L of 1% carrageenan was injected into their right hind paw to induce edema. The thickness of the paw was evaluated at 1, 2, and 4 h. At last, mice were euthanized and the paw tissues were collected and kept at −80°C for the next study. The inflammatory cytokines including IL 6 and TNF-*α* were measured using specific ELISA kits (R&D, Minneapolis, MN, USA). SOD activity and MDA content were investigated using special test kits (Nanjing Jiancheng Bioengineering Institute, China).

### 2.10. Statistical Analysis

All data were presented as means±S.D. Statistical significance was analyzed using one-way ANOVA with Tukey multiple comparison test applied GraphPad Prism 5 Software (GraphPad Software, San Diego, CA). *p* < 0.05 was recognized as statistically significant.

## 3. Results

### 3.1. UPLC-QTOF-MS/MS Analysis of SPE

In the present study, a qualitative analysis of the composition of SPE was performed using UPLC–DAD-ESI-QTOF-MS. The DAD chromatogram at 320 nm of SPE was shown in Figure 1. As listed in Table 1, twenty-four chemical compounds were identified in the SPE. The compounds of SPE were tentatively identified with the MS data and by comparing with published literatures [20–31]. In brief, these compounds including 7 phenolic acids and their derivatives, 17 flavonoids belonged to the groups of flavones, flavonols, and flavanones.

### 3.2. Effect of SPE on Viability of RAW 264.7 Macrophages

**Figure 2:**
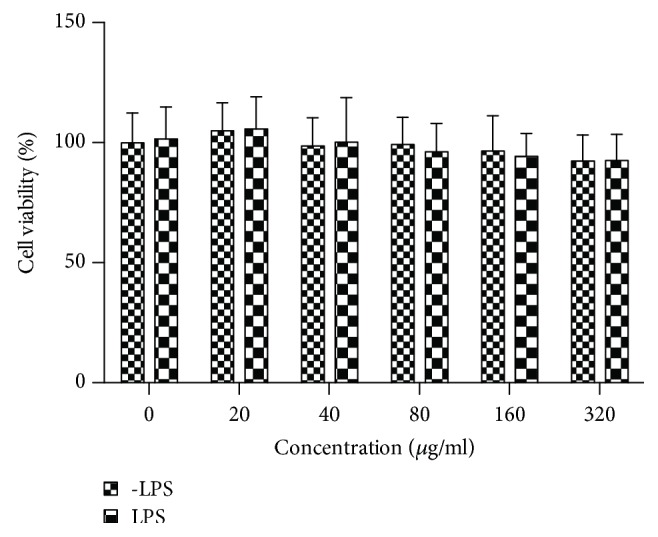
Effect of SPE on RAW 264.7 macrophage cell viability. Macrophages were cultured with SPE with or without LPS for 24 h, and cell viability was analyzed using the MTT assay.

MTT assay was performed to calculate the cellular cytotoxicity of SPE with or without LPS. The results indicated SPE with no cytotoxicity even at a high concentration (160 *μ*g/mL) on RAW 264.7 no matter the existence of LPS (Figure 2). Therefore, in this study 10, 20, 40, and 80 *μ*g/mL of SPE were selected for next study.

### 3.3. Effect of SPE on the Inflammatory Cytokine Production and Their Related Gene Expression

**Figure 3:**
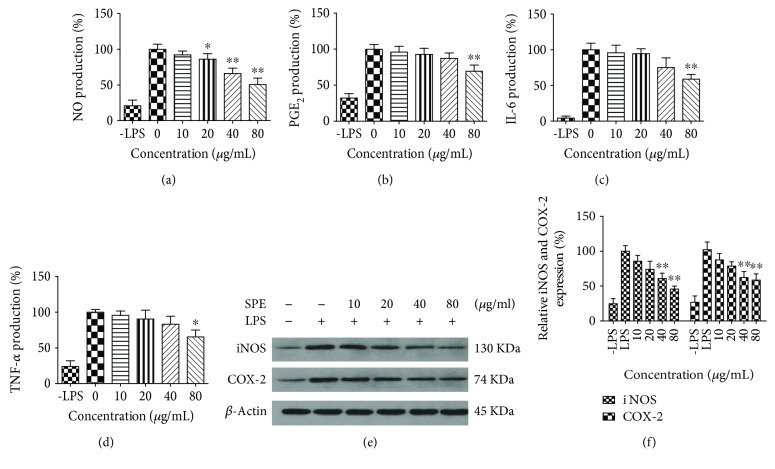
Influences of SPE on the production of proinflammatory cytokines and related protein expression. Mouse macrophages were induced with LPS in the presence of SPE for 16 h. Culture supernatant was removed and centrifuged to remove particulates and analyzed for production of these cytokines: (a) NO; (b) PGE_2_; (c) IL-6; and (d) TNF-*α*. (e) The expression levels of iNOS and COX2 were analyzed by western blot. (f) The band intensity of iNOS or COX2 was normalized with *β*-actin. ^∗^*p* < 0.05 and ^∗∗^*p* < 0.01 versus LPS alone group.

We first investigated whether SPE could inhibit the production of NO, which is the main proinflammatory mediator in LPS-induced inflammation in macrophages. As the results illustrated in Figure 3(a), LPS could induce large amount of NO; 40 *μ*g/mL of SPE could significantly reduce the NO production (*p* < 0.05) with an IC_50_ of 91.09 *μ*g/mL. For PGE_2_, similar to the NO production, with the increased concentration of SPE, the level of PGE_2_ was significantly decreased with an IC_50_ of 150.37 *μ*g/mL (Figure 3(b)). Similarly, treatment with SPE resulted in a concentration-dependent reduction of IL 6 and TNF-*α* with IC_50_ of 129.4 and 136.2 *μ*g/mL (Figures 3(c) and 3(d)). Further western blot assay (Figures 3(e) and 3(f)) also indicated that SPE showed significantly inhibitory effects on the expression of iNOS and COX-2.

### 3.4. Effects of SPE on Proinflammatory Cytokine Secretion

**Figure 4:**
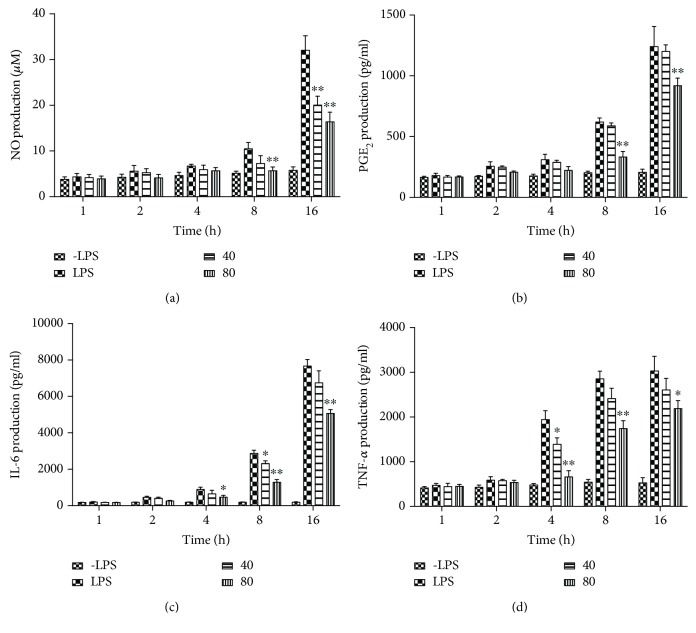
Influences of SPE on the secretion of proinflammatory cytokines with different LPS stimulation time. (a) NO; (b) PGE_2_; (c) IL-6; and (d) TNF-*α*. Cells were incubated with 1 *μ*g/ml LPS with the addition of 40 and 80 *μ*g/ml of SPE at 1, 2, 4, 8, and 16 h timepoints; culture supernatant was removed and centrifuged to remove particulates and analyzed for production of these cytokines. ^∗^*p* < 0.05 and ^∗∗^*p* < 0.01 versus LPS alone group.

To further investigate the influences of SPE on the secretion of specific cytokines stimulated by LPS in different treatment times, the contents of NO, IL-6, PGE_2_, and TNF-*α* were measured at 1, 2, 4, 8, and 16 h. As the results illustrated in Figure 4, with the induction of LPS, the productions of all of these four main proinflammatory cytokines were increasing with the time increased of LPS stimulation. 80 *μ*g/mL SPE could significantly inhibit the generation of these specific proinflammatory cytokines (*p* < 0.01) at 8 h and 16 h.

### 3.5. Effect of SPE on MAPK Phosphorylation and Activation of NF-*κ*B

**Figure 5:**
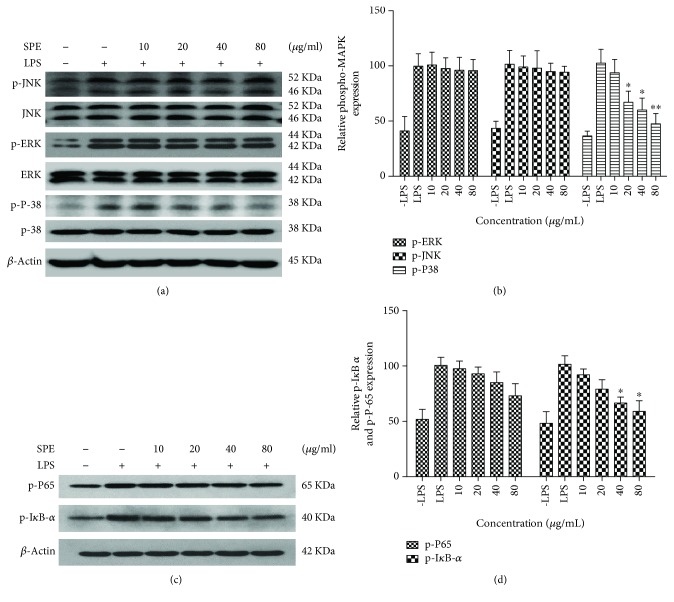
SPE inhibited the activation of LPS-induced MAPK (JNK, ERK, and p38) and NF-*κ*B (I*κ*B *α* and p65). Cells were incubated with 1 *μ*g/ml LPS with the addition of different concentration of SPE for 1 h; the protein were extracted and the phosphorylation of JNK, ERK, p 38, p 65, and I*κ*B *α* were analyzed with western blot assay. (a, c) Western blot assay of activation of MAPKs and NF-*κ*B were analyzed by and (b, d) The band intensity of phosphorylation MAPKs and NF-*κ*B were normalized with nonphosphorylation of MAPKs and *β*-actin. ^∗^*p* < 0.05 and ^∗∗^*p* < 0.01 versus LPS alone group.

**Figure 6:**
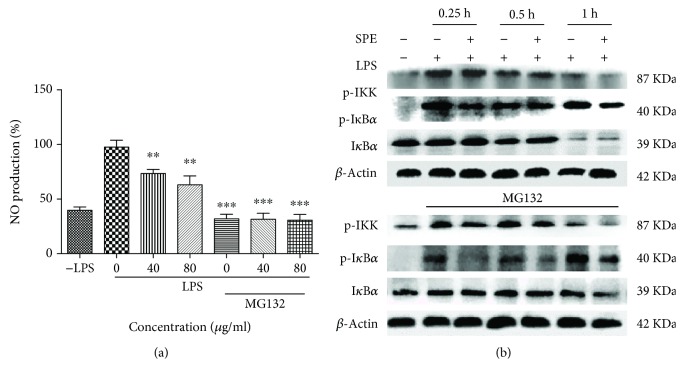
SPE reduced the activation of IKK and I*κ*B *α* and reversed the degradation of I*κ*B *α* during its inhibition of LPS-induced inflammation. (a) The NO production in the LPS induced RAW 264.7 cells with or without the treatment of SPE and MG 132. RAW 264.7 cells were first incubated with or without MG132 for 1 h and then treatment with LPS or LPS+SPE for 16 h. Culture supernatant was collected and NO production was measured using Griess reagent. (b) The phosphorylation of IKK and I*κ*B *α* in macrophages. Apart treatment with LPS and SPE, Raw 264.7 cell were also incubated with or without MG132, at 0.25 h, 0.5 h, and 1 h; the proteins were extracted and were analyzed using western blot assay. ^∗∗^*p* < 0.01 and ^∗∗∗^*p* < 0.001 versus LPS alone.

The NF-*κ*B and MAP kinase signaling (p38, JNK, and ERK) pathways regulate the LPS-induced inflammatory response and also played key roles in the occurrence and development of inflammation [32]. To further clarify the underlying mechanism of the anti-inflammatory ability, influences of SPE on activation of NF-*κ*B and phosphorylation of MAPKs were evaluated using western blot assays. As the data presented in Figures 5(a) and 5(b), SPE showed a significant reduction on the phosphorylation of p38. In this study, SB 203580, a p-38 MAPK inhibitor, also showed a significantly inhibitory activity on the NO production in LPS-induced RAW264.7 cells (32.06 ± 3.14 vs 18.51 ± 2.37 *μ*M). Furthermore, the phosphorylation of I*κ*B *α* and p65 appeared after LPS stimulation for 60 min (Figures 5(c) and 5(d)). The phosphorylation of p65 was significantly decreased with the treatment of SPE; the phosphorylation of I*κ*B-*α* also decreased with the treatment of 80 *μ*g/mL SPE even though there was no significant difference. These data indicated that the inhibitory effect of SPE on LPS-induced phosphorylation of p 38 MAPKs and activation of NF-*κ*B was partly associated with its anti-inflammatory potential. In this study, we also used MG132, a proteasome inhibitor, to clarify the effect of SPE. As the results illustrated in Figure 6, MG132 showed significantly inhibitory effect on the LPS-induced inflammatory (Figure 6(a)). And as the results showed in Figure 6(b), LPS could significantly induce the phosphorylation of IKK and I*κ*B and induce the degradation of I*κ*B *α*. However, SPE could reverse this to reduce the development of inflammatory process.

### 3.6. Antioxidative Activities of SPE

**Figure 7:**
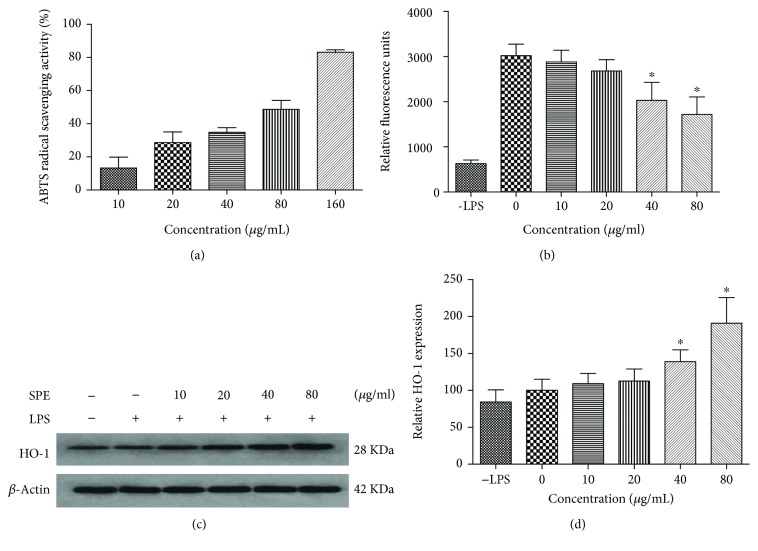
SPE showed strong antioxidative potential *in vitro* chemical and cell models. (a) The ABTS^+^ scavenging activity of SPE. (b) SPE inhibited ROS production in LPS-stimulated macrophages. (c, d) SPE enhanced the HO-1 expression. ^∗^*p* < 0.05 versus LPS alone group.

Previous studies have indicated that *in vitro* ABTS radical scavenging activity can potentially be used as marker for evaluating the anti-inflammatory activity of flavonoids [33]. Therefore, the antioxidant activity of SPE was firstly investigated using the ABTS assay. As the data presented in Figure 7(a), the ABTS radical scavenging activity of SPE increased with the increasing of SPE concentration with an EC_50_ value of 61.6 *μ*g/mL. At the concentration of 160 *μ*g/ml, about 80% of the ABTS free radical was scavenged. The Vc equivalent antioxidant capacity of SPE was calculated as 0.18 g per gram SPE.  Figure 7(b) showed that with the stimulation of LPS, the intracellular ROS were accumulated in RAW 264.7 cells, whereas SPE showed a strong inhibitory effect on the ROS production.

HO-1 has been reported as a stress-inducible protein induced by many stimuli such as oxidative stress and UV light [34, 35], and upregulation of the expression of HO-1 has been proved as a useful approach to improve oxidative injury and macrophage activation [35–38]. Therefore, the expression of HO-1 with SPE treatment was also evaluated in this study (Figures 7(c) and 7(d)). These results indicated that the expression of HO-1 was increased with the treatment of SPE.

### 3.7. *In Vivo* Anti-Inflammatory Activity of SPE

**Figure 8:**
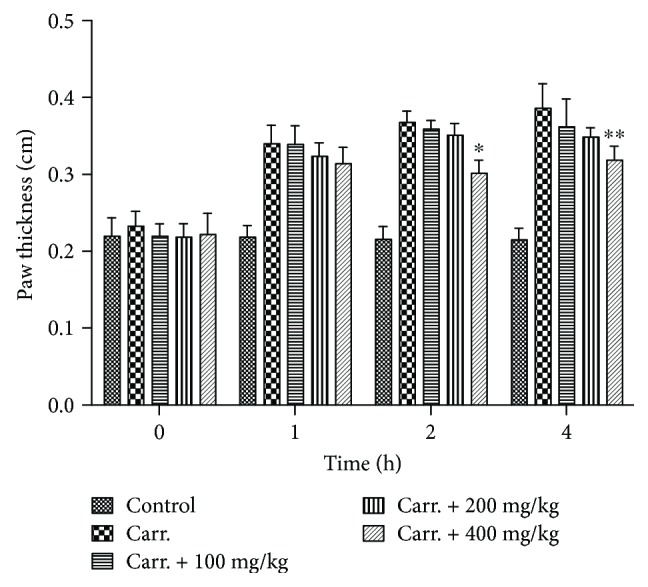
SPE inhibited the development of the paw edema induced by carrageenan injection. The edema model was induced with 30 *μ*L of 1% carrageenan. Different doses of SPE were administered orally 1 h prior to carrageenan injection. ^∗^*p* < 0.05 and ^∗∗^*p* < 0.01 versus carrageenan group.

**Figure 9:**
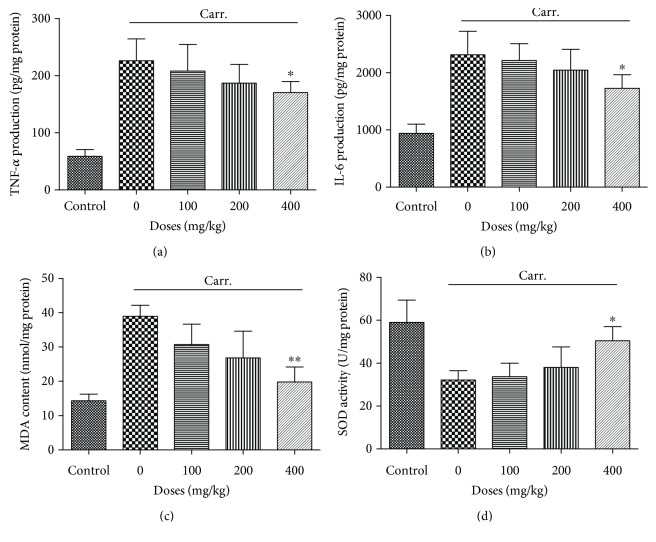
Influence of SPE on the production of inflammatory biomarkers and oxidative stress parameters in paw tissue. The edema was induced with 30 *μ*L of 1% carrageenan. Different doses of SPE were administered orally 1 h prior to carrageenan injection. (a) TNF-*α* expression levels. (b): IL-6 expression levels. (c) MDA level. (d) SOD activity in edema tissue. ^∗^*p* < 0.05 and ^∗∗^*p* < 0.01 versus model group.

The anti-inflammation potential of SPE was further investigated using an *in vivo* mouse paw edema model. As the data illustrated in Figure 8, the paw thickness significantly increased after the carrageenan injection, and 400 mg/kg of SPE showed a significantly inhibitory activity of paw edema (Figure 8). With the oral administration of 400 mg/kg of SPE, the paw thickness significantly decreased, which was 0.30 ± 0.02 and 0.32 ± 0.02 cm compared with 0.37 ± 0.01 and 0.39 ± 0.03 cm at 2 h and 4 h, respectively. For TNF-*α* and IL-6, a large amount of TNF-*α* and IL-6 were induced with the injection of carrageenan (Figures 9(a) and 9(b)); 400 mg/kg of SPE could significantly decrease the generation of TNF-*α* and IL-6, which was 170.23 ± 19.58 and 1728.21 ± 237.69 pg/mg protein compared with 226.01 ± 38.70 and 2314.41 ± 409.04 pg/mg protein (*p* < 0.05), respectively. In addition, 400 mg/kg of SPE also could significantly decrease the MDA content (19.82 ± 4.36 vs 39.71 ± 5.30 nmol/mg protein, *p* < 0.01) (Figure 9(c)). These *in vivo* results were also in accordance with the result from cell culture model. Meanwhile, SOD activity assay also indicated that the decrease of SOD activity by the carrageenan injection was reversed with the treatment of SPE (50.53 ± 6.59 vs 32.19 ± 4.28 U/mg protein) (Figure 9(d)).

## 4. Discussion

In recent years, the functional foods have received increasing attention worldwide. It can not only supply the nutrients but also provide many phytochemicals which play as a functional factor for human health, especially for these people with chronic disease or in the state of subhealth, such as chronic low-grade inflammation; these functional foods may be the best choice to improve their healthy rather than treatment with drugs [39, 40]. Previous studies have proved that shepherd's purse (*Capsella bursa-pastoris*) exerts multihealthy benefits, such as antimicrobial [41], anti-inflammatory [13], cardiovascular, reproductive, anticancer [16, 42], hepatoprotective, sedative, and other pharmacological effects [43]. Therefore, the chemical components of shepherd's purse ethanol extract were first characterized by UPLC-QTOF-MS/MS, and then the anti-inflammatory effects and its underlying mechanisms of SPE in LPS-induced RAW 264.7 inflammation model and *in vivo* mouse model were also systemically investigated in this study.

Macrophages play key roles in the immune system; the activation of macrophages induced the secretion of many inflammatory mediators, and also coupled with a high degree of oxidative stress [44]. SPE showed significantly inhibitory on the production of NO and PGE_2_. The overexpression of circulating inflammatory factors, including IL-6, IL-1*β*, TNF-*α*, and MCP-1, transforming growth factor (TGF)-a, TGF-b, and IFN-c also have been proved associated with low-grade, chronic inflammation [45, 46]. In this study, the results indicated that the production of TNF-*α* and IL-6 were significantly inhibited with the treatment of SPE from 4 h. These findings demonstrated that SPE could attenuate LPS-induced macrophage activation, which indicated that SPE possesses potential anti-inflammatory activity.

Inflammation is regulated by many proinflammatory mediators and cell signal pathways [47]. In this process, NF-*κ*B, MAPKs, and Nrf 2/HO-1 pathway have been proved that played key roles in mediating the inflammatory responses [19, 48–52]. Therefore, these pathways are potential targets for pharmacological intervention in the treatment of inflammation [49, 50]. This study found that SPE could inhibit the phosphorylation of p38 MAPK and reduce the subsequent inflammatory response. Similarly, NF-*κ*B is also an important signal pathway involved in immune and inflammatory responses [53, 54]. In addition, following treatment with SPE, LPS induced the phosphorylation of I*κ*B and P-65 was also inhibited in RAW 264.7 macrophages with the treatment of SPE, which indicated that SPE could prevent the activation of NF-*κ*B to exert its anti-inflammatory potential. Furthermore, there were very close connections between these two signal pathways. From the above results, we could find that both of the activation of p 38 MAPKs and NF-*κ*B signal pathways were blocked by the treatment of SPE. Therefore, these data suggested SPE exerts its anti-inflammatory potential at least partly associated with its regulation on the activation of MAPKs and NF-*κ*B pathways.

It has been proved that there was a connection between chronic inflammation and oxidative stress, and free radical-induced damage also could induce many chronic health problems [55–57]. In the inflammatory process, ROS have been proved to participate in the LPS-stimulated inflammation process by activating specific signaling pathways, resulting in the production of many specific cytokines [35, 57–60]. In addition, the upregulation of HO-1 was recognized as a pivotal response to different kinds of stress, it could exert its anti-inflammatory potential through inhibiting the excessive production of specific cytokines, and also through its regulation on macrophage switching to an M2-phenotype [61]. In this study, the data indicated SPE with strong antioxidative activity; meanwhile SPE could inhibit ROS production in macrophage cells whereas enhanced the expression of HO-1. These data proved the strong antioxidant activity also played an important role in the anti-inflammatory effect of SPE.

Carrageenan-induced paw edema animal model is usually applied to assess the different phases of inflammation reaction and evaluate the anti-inflammatory agents; it can induce acute inflammation, release of inflammatory mediators, and production of free radicals [62–64]. Therefore, this animal model was also used in this study. The results proved that SPE could inhibit the development of the carrageenan-induced edema, which was consisted with the *in vitro* findings. Meanwhile, the induction of inflammation by carrageenan was compared by generation of ROS and increased oxidative stress [64]. The animal study indicated that there was a significant increase of MDA content along with a distinct decrease of SOD activity with the injection of carrageenan in the model group. However, with the treatment of 400 mg/kg SPE, increase of SOD activity and the decrease of MDA in paw edema tissue were observed. These results indicated that SPE also showed strong anti-inflammatory and antioxidative potential *in vivo*.

In this study, about 24 chemical compounds were identified from the extracts of *Capsella bursa-pastoris*. As the HPLC-MS results, SPE contain a high amount of flavonoids, such as quercetin, kaempferol-7-O-rhamnopyranoside, quercetin-3-O-glucopyranoside, quercetin-6-C-glucopyranoside, and kaempferol-3-O-rutinoside; these results were also consistent with some previous findings [43, 65, 66]. Many studies had investigated the health benefits of these components, for example, antioxidative, ant-obesity, and anticancer activities [64, 67, 68]. Therefore, the flavonoid constituents presented in SPE could play key roles for its antioxidative and anti-inflammatory activity. However, since the extract contained many compounds, further works are still needed to be undertaken to investigate the anti-inflammatory properties of single compounds to further clear the anti-inflammatory potential of SPE.

## 5. Conclusion

In conclusion, in this study, the chemical composition, anti-inflammatory potential of shepherd's purse, and its underlying mechanisms were first systematically evaluated. Our findings indicated that *Capsella bursa-pastoris* (L.) Medik might reduce NO and PGE_2_ production and also inhibited the production of TNF-*α* and IL-6 in inflammatory development process. The underlying mechanism study proved that the anti-inflammatory potential of *Capsella bursa-pastoris* (L.) Medik was through the inhibition of the activation of p-38 MAPKs and NF-*κ*B pathways. Taken together, besides the good nutritional value and delicious taste already described in the previous studies, *Capsella bursa-pastoris* (L.) Medik also showed special health benefits, suggesting that it may be interesting not only for human health but also as food additive.

## Figures and Tables

**Table 1:** Identification of compounds in shepherd's purse extract by UPLC-QTOF-MS/MS.

No.	t_R_ (min)	[M-H]^−^ (m/z)	Major fragment ions (m/z)	Tentative identification	References
1	7.64	353.0882	191.0553	5-*O*-Caffeolyquinic acid	[21]
			160.8414		
			135.0285		

2	8.06	367.1030	193.0493	1-*O*-Feruloylquinic acid	[22]
			134.0362		

3	8.51	337.0929	191.0554	4-*p*-Coumaroylquinic acid	[23]
			173.0448		

4	8.77	337.0924	191.0552	5-*p*-Coumaroylquinic acid	[24]

5	8.91	337.0924	173.0450	3-*p*-Coumaroylquinic acid	[23]

6	9.07	163.0392	163.0386	*p*-Coumaric acid	[25]
			119.0492		

7	9.56	367.1028	298.0486	5-*O*-Feruloylquinic acid	[24]
			191.0551		
			173.0448		

8	9.60	579.1343	459.0821	Luteolin-6-*C*-pentoside-8-*C*-hexoside	[22]
			429.0771		
			357.0613		
			327.0504		
			309.0403		
			285.0396		

9	9.91	447.0930	357.0610	Luteolin-6-*O*-glucoside	[26]
			327.0508		
			298.0470		
			285.0399		
			269.0452		

10	10.15	563.1392	473.0954	Apigenin-6-*C*-hexoside-8-*C*-pentoside	[27]
			443.1048		
			383.0755		
			353.0662		

11	10.57	431.1925	293.0453	Kaempferol-*O*-rhamnoside	[28]
			284.0307		
			255.0252		

12	10.68	609.1446	463.0859	Quercetin-3-*O*-rutinoside	[29]
			301.0340		
			300.0273		
			271.0247		
			255.0301		

13	10.85	463.0882	300.0269	Quercetin-3-*O*-glucoside	[28]
			301.0331		
			271.0244		

14	11.01	593.1501	443.0973	Kaempferol-3-*O*-rutinoside	[30]
			323.0554		
			285.0405		

15	11.33	505.0986	300.0272	Quercetin-3-(6-*O*-acetyl-beta-glucoside)	[28]
			271.0244		
			255.0292		

16	11.499	593.1499	285.0396	Kaempferol-3-*O*-beta-D-rutinoside	[28]
			284.0320		
			255.0298		

17	11.62	725.1710	725.1678	Kaempferol triglycoside	[26]
			605.1277		
			429.0818		
			327.0488		
			309.0392		
			285.0387		

18	11.68	447.0938	447.0905	Kaempferol-*O*-glucoside	[28]
			429.0782		
			284.0313		
			255.0290		
			227.0341		

19	11.75	623.1604	531.0240	Isorhamnetin-3-rutinoside	[24]
			427.9770		
			315.0495		
			314.0418		
			271.0243		
			255.0283		

20	11.85	755.1802	635.1353	Diosmetin-7-*O*-triglycoside	[31]
			579.1293		
			429.0812		
			357.0258		
			309.0400		

21	11.93	623.1611	501.1388	Quercetin rhamnoside glucuronate	[32]
			447.1135		
			429.1028		
			337.0923		
			269.0453		

22	12.39	607.1674	551.1418	Chryseoriol- -rutinoside	[21]
			515.0217		
			429.1035		
			299.0561		
			284.0328		

23	13.00	461.1088	299.0558	Chryseoriol-7-*O*-glucoside	[23]
			284.0325		
			256.0376		
			116.9280		

24	14.04	299.1850	299.1852	Chrysoeriol	[29]
			284.0325		
			255.0302		

## Data Availability

The data used to support the findings of this study are available from the corresponding author upon request.
